# Smoking in early adulthood is prospectively associated with prescriptions of antipsychotics, mood stabilizers, antidepressants and anxiolytics

**DOI:** 10.1017/S0033291720005401

**Published:** 2022-10

**Authors:** Eline Borger Rognli, Jørgen Gustav Bramness, Tilmann von Soest

**Affiliations:** 1 Section for Clinical Addiction Research, Department on Substance Use Disorder Treatment, Oslo University Hospital, Oslo, Norway; 2 Norwegian Institute of Public Health, Oslo, Norway; 3 Institute for Clinical Medicine, UiT – The Arctic University of Norway, Tromsø, Norway; 4 Norwegian National Advisory Unit on Concurrent Substance Abuse and Mental Health Disorders, Innlandet Hospital Trust, Hamar, Norway; 5 Department of Psychology, PROMENTA Research Center, University of Oslo, Oslo, Norway; 6 Norwegian Social Research (NOVA), OsloMet – Oslo Metropolitan University, Oslo, Norway

**Keywords:** Affective disorders, anxiety disorder, bipolar disorder, cigarette smoking, drug prescriptions, mental disorders, nicotine dependence, pharmacoepidemiology, psychotic disorders

## Abstract

**Background:**

Whether smoking should be regarded as a risk factor for mental disorders remains unresolved. Prescribed psychotropic drugs can be used as indications for mental disorders. We investigated how smoking was prospectively related to prescription of antipsychotics, mood stabilizers, antidepressants, and anxiolytics.

**Methods:**

Information about smoking, including the Fagerström Test for Nicotine Dependence, and relevant confounders, were obtained from the population-based Young in Norway Study (*N* = 2602), with four data collection waves between 1992 and 2006. These survey data were linked with information on prescriptions for psychotropic drugs from the comprehensive, nationwide Norwegian Prescription Database from 2007 to 2015.

**Results:**

Daily smoking with high dependence in 2006 at age 28.5 (s.d. = 2.0) was associated with filling prescriptions of antipsychotics (OR, 6.57, 95% CI 2.19–19.70, *p* = 0.001), mood stabilizers (OR, 7.11, 95% CI 2.51–20.15, *p* < 0.001) and antidepressants (OR, 1.91, 95% CI 1.13–3.23, *p* = 0.016) 1–9 years later. Associations remained significant after adjustment for a variety of potential confounders measured before the assessment of smoking, including sociodemographic background, conduct problems, cannabis use, mental distress, and previous prescriptions for psychotropic medications. The association between smoking and prescription of anxiolytics was weaker and more unstable.

**Conclusions:**

In this study of young adults, daily smoking with high dependence was associated with later prescriptions of antipsychotics, mood stabilizers and antidepressants, indicating smoking as a risk factor for mental disorders treated with these drugs.

## Introduction

Cigarette smoking is the leading cause of preventable death, with well-established causal associations to several somatic diseases (Reitsma et al., 2017). Mental disorders are also associated with smoking, in the sense that persons with mental disorders more often smoke compared to persons without mental disorders (Lasser et al., 2000). Whether smoking may be a risk factor for mental disorders, and the magnitude of this potential risk, is less clear.

Smoking prevalence among persons with schizophrenia is high, with a five-fold risk for patients with schizophrenia to smoke compared to non-patients (De Leon & Diaz, 2005; Salokangas, Honkonen, Stengård, Koivisto, & Hietala, 2006). Some research suggests that as many as 60% of patients with schizophrenia smoke and that one-third smoke in the excess of 20 cigarettes per day (Salokangas et al., 2006). The high prevalence and intensity of smoking have long been seen as the patient's attempt to alleviate symptoms (Jacobsen et al., 2004; Sacco et al., 2005) and counter side-effects of medications (Goff, Henderson, & Amico, 1992). A benchmark study by Andréasson, Engström, Allebeck, and Rydberg (1987) found that cannabis, but not smoking, was related to later development of schizophrenia. In contrast, two recent meta-analyses of prospective studies suggest that smoking may be a risk factor for psychosis, with a doubled relative risk of developing schizophrenia for smokers compared to non-smokers and with heavier smoking associated with greater risk (Gurillo, Jauhar, Murray, & MacCabe, 2015; Hunter, Murray, Asher, & Leonardi-Bee, 2020). However, the number of longitudinal studies addressing the issue is limited, and the two meta-analyses were each based on only five individual studies, of which four were the same in both meta-analyses (Kendler, Lönn, Sundquist, & Sundquist, 2015; Sørensen, Mortensen, Reinisch, & Mednick, 2011; Weiser et al., 2004; Zammit et al., 2003).

Smoking is two to three times more common for persons with bipolar disorder compared to the general population (Heffner, Strawn, DelBello, Strakowski, & Anthenelli, 2011), and smoking prevalence in this patient group is higher than for patients with major depression but lower than for patients with schizophrenia (Jackson, Diaz, Lopez, & de Leon, 2015). Despite these high rates, few prospective studies investigate smoking as a potential risk factor for bipolar disorder. Smoking may play a mediating role in the severity of bipolar disorder (Thomson et al., 2015), and nicotine dependence and bipolar disorder were found in one study to predict the onset of each other (Martínez-Ortega et al., 2013). Also, a recent Mendelian randomization study concluded that smoking can be viewed as a causal risk factor for developing bipolar disorder (Vermeulen et al., 2021).

The relationship between smoking and depression and anxiety seems to be bidirectional (Fluharty, Taylor, Grabski, & Munafò, 2016). There is evidence that depression increases the risk of smoking (Khaled, Bulloch, Williams, Lavorato, & Patten, 2011), whilst others report that smoking increases the risk of depression (Boden, Fergusson, & Horwood, 2010; Pasco et al., 2008). Meta-analyses find that smokers have greater odds for follow-up incidents of depression compared to non-smokers, both when including studies of adults (Luger, Suls, & Vander Weg, 2014) and adolescents (Chaiton, Cohen, O'Loughlin, & Rehm, 2009). Studies on anxiety show the same pattern: some find that anxiety may increase the risk of later smoking (Senol, Donmez, Turkay, & Aktekin, 2006) while other shows that smoking is a risk factor for the development and worsening symptoms of anxiety (Cuijpers, Smit, Ten Have, & De Graaf, 2007; Moylan et al., 2013; Mykletun, Overland, Aarø, Liabø, & Stewart, 2008; Okeke, Spitz, Forman, & Wilkinson, 2013; Taylor et al., 2014).

Comparison of the risk imposed by smoking on different types of mental disorders is difficult, as there are few longitudinal studies in this area for some disorders, and single studies typically look at only one of the mental health outcomes. The studies differ with regard to operationalization of exposure and outcome and to which extent they adjust for potential confounders. Studies combining several mental health outcomes using the same methodology would add valuable information. Also, as smoking behavior is diverse and the risk of some mental disorders may be higher for the heavy smokers (Hunter et al., 2020), a differentiation between daily smoking with and without nicotine dependence is recommended (Gurillo et al., 2015).

In this prospective longitudinal study, we investigated the relationships between smoking and later prescription of antipsychotics, mood stabilizers, antidepressants and anxiolytics. We differentiated between previous smoking and daily smoking with a high and low degree of nicotine dependence. We included baseline measurement of a multitude of possible confounders, such as several variables of socio-demographic background, parental smoking, conduct problems, mental distress and use of cannabis. To decrease the possibility of reversed causality, we finally adjusted for prescriptions of psychotropic medications the year before smoking was assessed.

## Methods

### Procedure and participants

The study was based on data from the Young in Norway study, described in more detail elsewhere (von Soest, Bramness, Pedersen, & Wichstrøm, 2012; von Soest, Wichstrøm, & Kvalem, 2016). In short, the initial sample at T_1_ (1992) was composed of students in grades 7–12 drawn from 67 junior and senior high schools in Norway (age span 12–20 years, response rate 97%). The only exclusion criterion was a severe lack of reading capability. Students were followed up in 1994 (T_2_), 1999 (T_3_), and 2005–2006 (T_4_), with the mean age of the participants across the four data collection waves being: T_1_: 15.1 (s.d. = 2.0 years), T_2_: 16.5, T_3_: 23.0 and T_4_: 28.5 years. The cumulative response rate across all four waves was 69%.

At the last data collection, respondents were asked for their consent to link the data to several registers, to which 2602 respondents (90%) agreed. These 2602 respondents, 1145 men (44%) and 1457 women (56%), who completed the assessment at T_4_ and consented to registry linkage, constitute the sample in the present study.

Information about prescriptions for mental disorders was drawn from the Norwegian Prescription Database (NorPD). This registry is administered by the Norwegian Institute of Public Health and contains information on all prescriptions dispatched to pharmacies outside of institutions, prescribed to individuals, from 2004. The database includes information about the date of prescription filling, anatomical-therapeutic-chemical (ATC) code of the drug (WHO, 2019), and the number of daily defined doses. The outcome in the current study included data from the NorPD from 1 January 2007 to 31 December 2015; 1–9 years after T_4_.

Attrition analysis showed that male gender (OR 1.27, 95% CI 1.15–1.40, *p* < 0.001), having parents with non-Norwegian countries of origin (OR 1.66, 95% CI 1.28–2.15, *p* < 0.001), low parental education (OR 0.86, 95% CI 0.82–0.90, *p* < 0.001), not growing up with both biological parents (OR 0.81, 95% CI 0.73–0.90, *p* < 0.001), conduct problems at T_1_ (OR 1.66, 95% CI 1.45–1.90, *p* < 0.001), and alcohol intoxication at T_1_ (OR 1.05, 95% CI 1.01–1.09; *p* = 0.02) were significantly related to dropout, whereas parental smoking and mental distress at T_1_ were not (*p* > 0.05).

### Measures

#### Smoking

Smoking history was assessed in 2005–2006 (T_4_) by asking respondents whether they currently smoked daily or had done so earlier. To examine the adequacy of the prevalence estimates of daily smoking, we compared the data with independently obtained official national estimates from Statistics Norway (Statistics Norway, 2020). These comparisons showed high agreement, with a slightly higher prevalence rate for our data set (23% *v.* 20%) in the relevant age groups, which may relate to the lower response (61%) in the national data set. The Fagerström Test for Nicotine Dependence (FTND; Fagerström and Schneider, 1989; Heatherton, Kozlowski, Frecker, and Fagerström, 1991) was included in the questionnaire at T_4_ to assess nicotine dependence by self-report. The scores range from 0 to 10 (Cronbach's *α* = 0.68, based on observations from all respondents who smoked at T_4_; *n* = 517), and the instrument has satisfactory reliability and validity (Pomerleau, Carton, Lutzke, Flessland, & Pomerleau, 1994). In most analyses we used a dichotomized measure, where scores of 4 or higher is considered to indicate nicotine dependence (Huang, Lin, & Wang, 2008). Based on self-reported smoking and the FTND at T_4_, we divided the material into four categories: Those who had never smoked daily; those who had smoked daily at least once in their lifetime, but not in the last 12 months; those who smoked daily with low dependence (FTDN <4); and those who smoked daily with high dependence (FTDN ⩾4).

#### Prescription of psychotropic drugs

Subjects were categorized according to ATC-codes in mutually exclusive groups as receiving antipsychotics, mood stabilizers, antidepressants or anxiolytics during the 9-year period from 2007 to 2015. A fifth group was created to capture prescriptions that due to dose and type of drug were evaluated to be psychotropic drugs used for other conditions than a mental disorder (mainly nausea, sleep problems or epilepsy). In addition to obtaining prescription data as an outcome from the 9-year period from 2007 to 2015, prescription data were also obtained from 2004, the year before smoking was assessed. The reason for the 1–9 years duration of the follow-up period was that the prescription data used in this study contained information up until 2015.

Hierarchical decision rules were used for assigning a prescription category when persons had received medications from more than one category. The hierarchy of decision rules was based on the fact that some types of medications are almost exclusively used for the treatment of a specific psychiatric disorder, such as lithium for bipolar disorder, independent of additional prescription of other types of psychotropic drugs. These psychotropic drugs were placed on the top in the decision hierarchy. In contrast, other medications may be used for the treatment of a specific psychiatric disorder only when not used in combination with other drugs. For example, antidepressants prescription may indicate treatment for unipolar depression, but only when not used in combination with lithium, as this would indicate the treatment of bipolar disorder. These types of medications were placed towards the bottom of the decision hierarchy. As a result, decision rules for assigning a prescription category were hierarchical prioritizing in the following order: (1) mood stabilizers, (2) antipsychotics, (3) antidepressants, (4) anxiolytics and (5) psychotropic drugs prescribed on other indications. To exemplify, persons with prescriptions of both antidepressants and mood stabilizers would have been categorized as receiving mood stabilizers. Some exceptions from the general rules were made to account for particular pharmacological combinations, such as only low-dose prescriptions and cases of only one prescription. The categorization principles were constructed without knowledge about smoking status, with the purpose to optimize the prescription categories as proxies for underlying mental disorders. A detailed description of the principles is provided in the supplement.

#### Socio-demographics

Age, gender and country of birth (Norway or other) were assessed at T_1_. Parental education was assessed at the same time point and was classified into five levels from *up to 9 years of basic education* (1) to *more than 3 years of university education* (5) for the parent with the highest education. We also asked whether the respondent was living with both biological parents or not at T_1_. At T_3_, respondents were asked whether their father and mother smoked daily during their childhood or not, and we contrasted those who had at least one parent who smoked in childhood with those who had no smoking parent.

#### Conduct problems and use of alcohol and cannabis

We used a 15-item measure of conduct problems at T_1_, which approximates diagnostic criteria for conduct disorder in the DSM-III-R (Wichstrom, Skogen, & Øia, 1996). Response options ranged from 1 (*never*) to 6 (*more than 50 times*). Mean scores across all items were computed (Cronbach's *α* = 0.75, based on *n* = 2367 observations). We assessed the number of alcohol intoxication episodes at T_1_ by asking how often respondents had drunken so much that they felt clearly intoxicated during the previous 12 months. Response options ranged from 1 (*never*) to 6 (*more than 50 times*). We also assessed at T_4_ whether respondents had used cannabis at least once the previous 12 months or not.

#### Mental distress

Mental distress was measured at T_1_ by 12 items from the Hopkins Symptom Checklist (Derogatis, Lipman, Rickels, Uhlenhuth, & Covi, 1974). The measure asks for ratings of symptoms of depression and anxiety the preceding week and applies a 4-point scale with the response options from 1 (*not bothered at all*) to 4 (*extremely bothered*). The items have been used in several studies to measure mental distress and have favorable psychometric properties (Pedersen & von Soest, 2009). Mean scores were computed and internal consistency was high (Cronbach's *α* = 0.85, based on *n* = 2397 observations).

### Analyses

Multinomial logistic regression analysis was conducted with prescription as dependent variable and smoking as the independent variable. The categories of ‘antipsychotics’, ‘mood stabilizers’, ‘antidepressants’, ‘anxiolytics’ and ‘psychotropic drugs on other indications’ were all compared to the reference category ‘no psychotropic drugs’. We compared levels of nicotine use (‘smoked daily before but not now’, ‘daily smoking, low dependence’, ‘daily smoking, high dependence’) with the reference category ‘never smoked daily’. We adjusted for potential confounders in four steps by including several indicators of socio-demographic background and parental smoking behavior in a first step, adding conduct problems and cannabis use in a second step, and adding mental distress in the third step as covariates. Moreover, in a fourth step, we additionally added a dummy variable indicating whether or not respondents had received prescriptions of psychotropic drugs in 2004 (*n* = 134), the year before information about smoking was obtained, thereby controlling for the possibility of prescriptions of psychotropic drugs preceding smoking.

A robust maximum likelihood estimator (MLR) was used in all analyses, thereby accounting for potential multivariate non-normality (Muthén & Muthén, 2012). Because respondents were originally recruited from 67 different schools, potential non-independence of observations owing to school clusters was addressed by estimating parameters by maximizing a weighted log-likelihood function, whereas standard error estimations were performed with a sandwich estimator. Missing data were handled by means of full information maximum likelihood estimation, thereby providing missing data routines that are considered to be state of the art (Muthén & Muthén, 2012; Schafer & Graham, 2002). The statistical program Mplus 7.4 was used for all regression analyses.

## Results

Of the total sample of 2602 individuals, 1468 (56.7%) had never been daily smokers, 606 (23.4) were former daily smokers, 305 (11.8%) were daily smokers with low dependence and 212 (8.2%) were daily smokers with high dependence. Data on smoking was missing for 11 participants. Based on the dominating type of prescriptions during the 9 year follow-up period, 33 individuals (1.3%) were categorized as receivers of antipsychotics, 36 individuals (1.4%) as mood stabilizer users, 233 individuals (9.0%) were prescribed antidepressants, 102 individuals (3.9%) received anxiolytics and 84 persons (3.2%) had been prescribed psychotropic drugs for presumably other reasons than a mental disorder (Table 1). The number of individuals who had not received any prescriptions of psychotropic drugs was 2114 (81.4%).

**Table 1.** Smoking, socio-demographics, conduct problems, drug use and mental distress according to filling prescriptions for psychotropic drugs

		No psychotropic drugs	Anti-psychotics	Mood-stabilizers	Anti-depressants	Anxiolytics	Psychotropic drugs on other indication	Difference test^a^	
		*n* = 2114 (81.2%)	*n* = 33 (1.3%)	*n* = 36 (1.4%)	*n* = 233 (9.0%)	*n* = 102 (3.9%)	*n* = 84 (3.2%)	χ*^2^*/*F*	*p*
Smoking									
Never smoked daily (T_4_)	*n* (%)	1273 (60.4%)	9 (27.3%)	10 (27.8%)	94 (40.5%)	44 (43.6%)	38 (45.8%)		
Smoked daily before but not now (T_4_)	*n* (%)	475 (22.6%)	7 (21.2%)	7 (19.4%)	64 (27.6%)	27 (26.7%)	26 (31.3%)		
Daily smoking, low dependence (T_4_)	*n* (%)	227 (10.8%)	6 (18.2%)	8 (22.2%)	39 (16.8%)	18 (17.8%)	7 (8.4%)		
Daily smoking, high dependence (T_4_)	*n* (%)	131 (6.2%)	11 (33.3%)	11 (30.6%)	35 (15.1%)	12 (11.9%)	12 (14.5%)		
Socio-demographics									
Female gender (T_1_)	*n* (%)	1137 (53.8%)	15 (45.5%)	25 (69.4%)	154 (66.1%)	69 (67.6%)	57 (67.9%)	28.39	<0.001
Age (T_1_)	Mean (s.d.)	15.24 (1.87)	14.88 (3.18)	15.14 (3.04)	15.28 (2.15)	15.37 (1.92)	15.37 (2.41)	0.43	0.828
Born in Norway (T_1_)	*n* (%)	1925 (97.0%)	27 (93.1%)	32 (94.1%)	214 (96.8%)	94 (96.9%)	77 (98.7%)	3.27	0.659
Parental education (T_1_)	Mean (s.d.)	3.42 (1.12)	3.08 (1.13)	3.33 (1.18)	3.16 (1.07)	3.33 (1.12)	3.21 (1.10)	2.60	0.024
Living with both parents (T_1_)	*n* (%)	1450 (71.5%)	20 (64.5%)	24 (70.6%)	130 (57.5%)	56 (55.4%)	56 (70.0%)	28.74	<0.001
One or both parents smoked (T_3_)	*n* (%)	849 (46.1%)	16 (64.0%)	20 (58.8%)	125 (60.5%)	46 (50.5%)	36 (48.0%)	23.74	<0.001
Conduct problems and drug use									
Conduct problems (T_1_)	Mean (s.d.)	1.34 (0.37)	1.36 (0.35)	1.37 (0.46)	1.41 (0.40)	1.45 (0.53)	1.41 (0.45)	2.71	0.019
Alcohol intoxication (T_1_)	Mean (s.d.)	1.88 (1.42)	1.54 (1.10)	1.57 (1.25)	2.11 (1.46)	2.10 (1.61)	2.09 (1.47)	2.29	0.043
Cannabis use at (T_4_)	*n* (%)	220 (10.6%)	13 (41.9%)	11 (33.3%)	43 (18.9%)	17 (17.0%)	13 (15.7%)	55.48	<0.001
Mental health and previous prescriptions									
Mental distress (T_1_)	Mean (s.d.)	1.57 (0.45)	1.70 (0.55)	1.65 (0.48)	1.80 (0.53)	1.64 (0.44)	1.65 (0.45)	10.75	<0.001
Psychotropic drug prescription in 2004	*n* (%)	40 (1.9%)	21 (36.4%)	19 (52.8%)	42 (18.0%)	10 (9.8%)	11 (13.1%)	373.40	<0.001

a Difference tests conducted by means of χ^2^ (categorical variables) and analysis of variance (continuous variables).

A total of 187 individuals had during the 9-year period received prescriptions from more than one prescription category. Among these, 129 persons had received a prescription of two prescription categories, 44 persons had received prescriptions from three prescription categories and 14 persons had received prescriptions from all four prescription categories. Information concerning the types and number of prescriptions of multiple categories of psychotropic drugs is provided in online Supplementary Tables S1 and S2.

Those who had not filled prescriptions for any psychotropic drugs during follow-up smoked less, had less often parents who smoked and reported less use of cannabis and lower mental distress, compared to those with some type of prescription of psychotropic medication (Table 1). There were differences between the smoking categories on all variables except being born in Norway, with a pattern of most healthy and protective background among those who had never been daily smokers and most unfavorable background among the daily smokers with high dependence (Table 2).

**Table 2.** Socio-demographics, conduct problems, drug use and mental distress according to smoking

		Never smoked daily	Smoked daily before but not now	Daily smoking, low dependence	Daily smoking, high dependence	Difference test^a^	
		*n* = 1468 (56.7%)	606 (23.4%)	*n* = 305 (11.8%)	*n* = 212 (8.2%)	χ^2^/*F*	*p*
Socio-demographics							
Female gender (T_1_)	*n* (%)	787 (53.6%)	366 (60.4%)	192 (63.0%)	107 (50.5%)	16.77	0.001
Age (T_1_)	Mean (s.d.)	15.26 (1.81)	15.42 (2.15)	14.95 (1.97)	15.04 (2.27)	4.66	0.003
Born in Norway (T_1_)	*n* (%)	1336 (97.1%)	551 (96.7%)	281 (96.6%)	190 (97.4%)	0.55	0.907
Parental education (T_1_)	Mean (s.d.)	3.43 (1.10)	3.38 (1.14)	3.38 (1.09)	3.02 (1.17)	6.26	<0.001
Living with both parents (T_1_)	*n* (%)	1077 (75.8%)	346 (60.6%)	205 (69.7%)	100 (48.8%)	89.67	<0.001
One or both parents smoked (T_3_)	*n* (%)	536 (41.4%)	267 (50.8%)	154 (59.7%)	132 (73.3%)	84.37	<0.001
Conduct problems and drug use							
Conduct problems (T_1_)	Mean (s.d.)	1.27 (0.30)	1.46 (0.46)	1.46 (0.42)	1.50 (0.51)	51.38	<0.001
Alcohol intoxication (T_1_)	Mean (s.d.)	1.63 (1.22)	2.31 (1.62)	2.20 (1.53)	2.31 (1.59)	42.27	<0.001
Cannabis use (T_4_)	*n* (%)	101 (7.0%)	86 (14.6%)	59 (20.1%)	14 (7.7%)	44.73	<0.001
Mental health and previous prescriptions							
Mental distress (T_1_)	Mean (s.d.)	1.55 (0.43)	1.64 (0.49)	1.65 (0.48)	1.73 (0.54)	13.69	<0.001
Psychotropic drug prescription in 2004	*n* (%)	54 (3.7%)	31 (5.1%)	16 (5.2%)	33 (15.6%)	53.39	<0.001

a Difference tests conducted by means of χ^2^ (categorical variables) and analysis of variance (continuous variables).

The unadjusted results of the multinomial regression analyses showed a strong association between daily smoking with high dependence and later prescription of antipsychotics and mood stabilizers, and a significant but weaker association for daily smoking with low dependence and antipsychotics and mood stabilizers (Table 3). The associations between daily smoking and antidepressants and anxiolytics were also significant, but with smaller effect sizes than those found for the association with antipsychotics and mood stabilizers.

**Table 3.** Results of multinomial regression analyses with the prescription of psychotropic drugs as an outcome

	Antipsychotics			Mood stabilizers			Antidepressants			Anxiolytics			Psychotropic drugs on other indications		
	*n* = 33			*n* = 36			*n* = 233			*n* = 102			*n* = 84		
	OR	95% CI	*p*	OR	95% CI	*p*	OR	95% CI	*p*	OR	95% CI	*p*	OR	95% CI	*p*
*Baseline Model: Unadjusted associations*															
Smoking															
Never smoked daily	(reference)			(reference)			(reference)			(reference)			(reference)		
Smoked daily before but not now	2.08	0.86–5.08	0.106	1.88	0.58–6.03	0.292	1.82	1.34–2.48	<0.001	1.64	0.95–2.84	0.075	1.83	1.14–2.95	0.013
Daily smoking, low dependence	3.74	1.16–12.10	0.028	4.49	1.58–12.79	0.005	2.33	1.68–3.22	<0.001	2.29	1.30–4.04	0.004	1.03	0.50–2.12	0.930
Daily smoking, high dependence	11.90	4.88–29.06	<0.001	10.71	4.14–27.69	<0.001	3.62	2.34–5.60	<0.001	2.65	1.42–4.96	0.002	3.07	1.71–5.53	<0.001
*Model 1: Adjustment for socio-demographics*															
Smoking															
Never smoked daily	(reference)			(reference)			(reference)			(reference)			(reference)		
Smoked daily before but not now	2.09	0.85–5.17	0.109	1.85	0.54–6.41	0.330	1.61	1.17–2.20	0.003	1.45	0.82–2.58	0.205	1.80	1.13–2.84	0.013
Daily smoking, low dependence	3.53	1.03–12.14	0.045	4.21	1.44–2.29	0.008	2.05	1.47–2.86	<0.001	2.17	1.23–3.80	0.007	1.01	0.48–2.12	0.978
Daily smoking, high dependence	10.28	4.04–26.19	<0.001	11.60	4.06–3.12	<0.001	2.84	1.75–4.60	<0.001	2.34	1.21–4.54	0.012	3.18	1.78–5.67	<0.001
*Model 2: Additional adjustment for conduct problems and drug use*															
Smoking															
Never smoked daily	(reference)			(reference)			(reference)			(reference)			(reference)		
Smoked daily before but not now	2.19	0.83–5.75	0.113	1.94	0.60–6.28	0.268	1.50	1.06–2.11	0.020	1.31	0.71–2.42	0.394	1.63	1.01–2.63	0.046
Daily smoking, low dependence	3.59	1.06–12.14	0.040	4.21	1.36–13.05	0.013	1.85	1.33–2.58	<0.001	1.92	1.01–3.64	0.045	0.89	0.41–1.91	0.762
Daily smoking, high dependence	8.88	3.30–23.90	<0.001	10.07	3.63–27.90	<0.001	2.42	1.45–4.01	0.001	1.98	1.02–3.86	0.045	2.70	1.48–4.91	0.001
*Model 3: Additional adjustment for mental distress*															
Smoking															
Never smoked daily	(reference)			(reference)			(reference)			(reference)			(reference)		
Smoked daily before but not now	2.15	0.82–5.61	0.119	1.92	0.59–6.26	0.278	1.44	1.01–2.05	0.042	1.31	0.71–2.41	0.390	1.62	1.00–2.61	0.048
Daily smoking, low dependence	3.55	1.04–12.16	0.043	4.16	1.33–12.97	0.014	1.78	1.28–2.48	0.001	1.92	1.02–3.61	0.044	0.88	0.41–1.90	0.744
Daily smoking, high dependence	8.27	3.02–22.61	<0.001	9.84	3.56–27.19	<0.001	2.15	1.27–3.64	0.005	1.96	1.01–3.80	0.045	2.61	1.43–4.78	0.002
*Model 4: Additional adjustment for prescription of psychotropic drugs in 2004*															
Smoking															
Never smoked daily	(reference)			(reference)			(reference)			(reference)			(reference)		
Smoked daily before but not now	2.12	0.77–5.87	0.147	1.94	0.57–6.66	0.293	1.49	1.05–2.13	0.027	1.32	0.72–2.41	0.371	1.64	1.01–2.65	0.045
Daily smoking, low dependence	3.79	1.07–13.39	0.039	4.21	1.38–12.86	0.012	1.91	1.35–2.69	<0.001	1.96	1.04–3.70	0.038	0.91	0.43–1.96	0.817
Daily smoking, high dependence	6.57	2.19–19.70	0.001	7.11	2.51–20.15	<0.001	1.91	1.13–3.23	0.016	1.84	0.93–3.64	0.078	2.41	1.30–4.48	0.005

OR, Odds ratio; 95% CI, 95% confidence interval of OR.

Through the four steps of adjustment, the pattern remained the same, with the final model estimating significant association between daily smoking with high dependence and prescription of antipsychotics (OR = 6.57, 95% CI 2.19–19.70, *p* = 0.001), mood stabilizers (OR = 7.11, 95% CI 2.51–20.15, *p* < 0.001) and antidepressants (OR = 1.91, 95% CI 1.13–3.23, *p* = 0.016). The association between daily smoking and prescription of anxiolytics was significant in some but not all models, and in the final model, the association was only significant for smoking with low dependence.

Daily smoking with high dependence was associated with later prescription of psychotropic drugs on other indications through all four steps of adjustment, with an OR = 2.41 (95% CI 1.30–4.48, *p* = 0.005) in the final model.

Due to somewhat low numbers in some prescription categories, we conducted additional analyses where daily smokers with high and low dependence were merged into one category. A new set of multinomial regressions was performed with the new smoking variable as predictor and results are presented in online Supplementary Table S4. Comparable results to the results of high and low dependence in Table 3 were obtained, with odds ratios generally between the odds ratios for high and low dependence and with more narrow confidence intervals.

Eight individuals had only received prescriptions of valproic acid or lamotrigine. Based on dose and type of drug it was not viable to decide whether this medication was prescribed for epilepsy or bipolar disorder. The reported results are based on a conservative categorization, defining these cases as epilepsy and thus in the category ‘psychotropic drugs on other indication’. Additional analyses with these individuals categorized as mood stabilizers yielded results very similar to those reported in Table 3, with an OR = 5.87 (95% CI 2.41–14.28, *p* < 0.001) for the mood stabilizers category in the final model (results not reported in the table).

To obtain information about the suitability of the logistic regression analyses conducted in Table 3, we conducted Hosmer–Lemeshow goodness of fit tests. As these tests cannot easily be conducted for multinomial logistic regression analyses, we conducted simple logistic regression analyses for each prescription category where participants with prescriptions from this category were contrasted with all other participants. All five tests yielded non-significant *p*-values (*p* > 0.05), thereby indicating a satisfactory model fit. Moreover, when estimating Variance Inflation Factors for all independent variables all values showed to be below 2, thereby indicating no issues with multicollinearity. Finally, we estimated Cook's Distance measures to examine the possible influence of outliers on the results. We identified five respondents with values > 1 (see Hosmer, Lemeshow, and Sturdivant, 2013). However, excluding observations from these respondents from the analyses did not change the results substantially.

## Discussion

In this study, linking a population-based investigation with data from an administrative register, we found that daily smoking was prospectively associated with later prescriptions of all classes of psychotropic drugs. The associations were distinctly stronger for those who reported higher nicotine dependence. The associations were significant after adjustment for a series of covariates, including baseline mental distress, cannabis use and prescriptions for psychotropic drugs the year before smoking was assessed. The effect sizes were larger for antipsychotics and mood stabilizers than for the other drugs.

The prospective association between nicotine dependence and antipsychotic prescriptions found in this study is in line with the more recent understanding of smoking as a potential risk factor for schizophrenia (Gurillo et al., 2015; Hunter et al., 2020). The marked larger effect sizes for those with a high level of dependence relative to other smokers indicate a dose–response relationship, which supports the notion of a causal association between smoking and psychotic disorders (Scott et al., 2018; Wium-Andersen, Ørsted, & Nordestgaard, 2015). The sixfold increase in risk among the daily smokers with high dependence may have to do with capturing the heaviest smokers, and we know that persons with schizophrenia smoke excessively (Salokangas et al., 2006).

Though it has been suggested that smoking may contribute to the development of the bipolar disorder (Slyepchenko, Brunoni, McIntyre, Quevedo, & Carvalho, 2016), the evidence of smoking as a risk factor for bipolar disorder is still scarce (Martínez-Ortega et al., 2013; Vermeulen et al., 2021). It is a previously well-described observation that persons with bipolar disorders tend to smoke (Heffner et al., 2011; Lasser et al., 2000) and the association between smoking and mood stabilizers found in this study, with a sevenfold increase in risk after adjustment for all confounders, suggests that smoking may be a risk factor for bipolar disorder. This should encourage further investigations on the association between smoking and bipolar disorder.

The results of an association between nicotine dependence and prescriptions of antidepressants are in line with results from previous research about the relationship between smoking and depression (Chaiton et al., 2009; Luger et al., 2014), and of similar effect sizes. Also, a study on the same cohort as the one used in the present study, but without the linkage to the prescription database, found a prospective association between nicotine dependence and later symptoms of depression (Pedersen & von Soest, 2009). Our results demonstrated somewhat stronger effect sizes for those with high relative to those with low dependence in most models and similar patterns have been found when using a graded nicotine exposure (Flensborg-Madsen et al., 2011). However, results from Mendelian randomization studies provide conflicting results about the causal effect of smoking on depression (Köhler et al., 2018; Wium-Andersen et al., 2015; Wootton et al., 2020).

Smoking has previously been found to increase the risk of later development of certain specific anxiety disorders, such as panic disorder and generalized anxiety disorder (Moylan, Jacka, Pasco, & Berk, 2012). Our results indicate that daily smoking is associated with a later prescription of anxiolytics, but with relatively small and uncertain effect sizes.

The association between daily dependent smoking and prescriptions presumably on other indication than a mental disorder may point to the somatic and lifestyle conditions associated with smoking (Reitsma et al., 2017). This outcome category may contain many symptoms and maladies and may be a poor proxy for any specific underlying condition. It is therefore difficult to conclude about the importance of this result.

A study limitation is that we do not know how good a proxy our classification of prescriptions is for mental disorders. The proportion receiving treatment of those who have a mental disorder may not be equal across diagnostic categories, and the treatment coverage is probably highest for psychotic disorders (Kohn, Saxena, Levav, & Saraceno, 2004). The anxiolytics category may involve the crudest approximation, as benzodiazepines are also used to treat several other disorders not related to anxiety, and other drugs, such as antidepressants, are used to treat anxiety disorders. The criteria for classification in this paper were based on clinical knowledge on drug use, but as most drugs have several uses, each individual case was reviewed to increase the likelihood of correct approximations. We find it unlikely that erroneous categorization could explain a large share of the difference in risk found in the present study. Also, receiving a prescription of a psychotropic drug has been used as an indirect measure of mental disorders in several previous studies (McKenzie, Murray, & Booth, 2013; Mok et al., 2013; Rognli, Bramness, & von Soest, 2020; Wium-Andersen et al., 2015).

The prevalence of daily smoking in Norway has declined dramatically during the last 50 years and is now just below 10% (Statistics Norway, 2020). The current data on smoking were obtained in 2006, at a time when this behavior was viewed as increasingly more unacceptable and stigmatized (Stuber, Galea, & Link, 2008). The change in smoking towards a more marginal phenomenon may imply more social disadvantage among smokers relative to non-smokers (Hiscock, Bauld, Amos, Fidler, & Munafò, 2012). Though we adjusted for socioeconomic background defined as parental education, we acknowledge that some of the effect of smoking on the different psychotropic outcomes could be an effect of residual confounding in terms of unmeasured social hardship. Also, we could not assess changes in smoking during follow-up and cannot rule out the possibility that mental disorders were triggered by smoking cessation.

As the attrition analyses showed, variables such as conduct problems and alcohol intoxication at T_1_ and low parental education were associated with dropout, meaning that our sample defined at T_4_ may have been a selection of the more privileged and untroubled youth. It is unlikely that this has affected the association between smoking and later psychotropic prescriptions in a substantial way.

We tried to reduce the probability of reversed causality by adjusting for early mental distress and previous prescriptions of psychotropic drugs, but such efforts may still be insufficient. It is well known that some mental disorders develop slowly, and the prodromal phase for psychosis may last for years (Yung & McGorry, 1996). Smoking may have been an attempt to regulate emotional distress among vulnerable individuals even long before the manifestation, or at least medication, of a mental disorder (Gehricke et al., 2007; Khantzian, 1997).

Despite these limitations, there is an advantage of using this prescription database, in that, all treated patients, from both primary health care and specialized health care are included. Norway is a country with a wide-ranging publicly financed health care system, and access to health care and prescriptions is based on illness severity, not on the private economy (Barber et al., 2017; Nordic Medico-Statistical Committee, 2017).

The FTND is an established measure of dependence and allows for a differentiation of the level of dependence among daily smokers (Heatherton et al., 1991). The increase in effect size for smokers with high dependence compared to other smokers, seen particularly pronounced for mood stabilizers and antipsychotics but also for antidepressants and anxiolytics, can be understood as a dose–response relationship, which is crucial for inferring causality (Hill, 1965).

This study is informative as it has a relatively large population-based sample, includes numerous relevant confounders, has a graded measure of nicotine dependence, and expands the knowledge from previous studies by using prescription data as an outcome. It is also an advantage that the study includes several types of mental health outcomes, allowing for comparison across different disorders. Though causal inference cannot be made based on observational data, the results indicate that smoking increases the risk of psychosis, bipolar disorder and depression, and perhaps also anxiety. The harmful effect of smoking not only on physical health but also on mental health should encourage mental health services to encompass health habits such as smoking in their treatment approach.

## Supplementary material

For supplementary material accompanying this paper visit https://doi.org/10.1017/S0033291720005401. click here to view supplementary material
